# Cross-buckled structures for stretchable and compressible thin film silicon solar cells

**DOI:** 10.1038/s41598-017-08012-y

**Published:** 2017-08-08

**Authors:** Jiyoon Nam, Bowook Seo, Youngjoo Lee, Dong-Ho Kim, Sungjin Jo

**Affiliations:** 1 School of Architectural, Civil, Environmental, and Energy Engineering, Kyungpook National University, Daegu, 41566 Korea; 2 Advanced Functional Thin Films Department, Korea Institute of Material Science (KIMS), Changwon, 51508 Korea

## Abstract

Increasing interests in stretchable electronic devices have resulted in vigorous research activities, most of which are focused on structural configurations. Diverse structural configurations are available for stretchability, including stiff-island, serpentine, and buckled structures. With easily deformable shapes and simple fabrication processes, buckled structures have the potential to realize stretchability. However, conventional buckled structures exhibit stretchability only in a single-axis direction. In the present study, a new type of cross-buckled structure, which can overcome the limitations of conventional buckled structures is developed. The stretchable thin film solar cells with the cross-buckled structure showed stable mechanical and electrical characteristics under both stretching and compressing conditions. The cross-buckled structure for stretchable electronic devices is expected to broaden the fields of wearable electronics, stretchable displays, and biocompatible applications.

## Introduction

Increasing demand for high-performance applications such as wearable devices, bio-inspired electronics, and aerospace equipment has instigated rapid progress in the field of stretchable electronics^1–7^. Rendering stretchability to electronic devices involves accommodating for stretching, flexing, bending, and other deformations along one or more axes^8–11^. However, conventional active device materials, mostly metals and semiconductors, are too rigid and brittle to achieve the required stretchability in electronic devices^12, 13^. Therefore, most studies are focused on modifying the structural configuration to develop stretchable electronic devices using conventional active device materials.

In the field of stretchable electronics, a variety of structures has been employed, including serpentine, stiff-island, and buckled structures^14–16^. In particular, the buckled structure is a widely used configuration in stretchable devices and fabrication of buckled structure is well-established^17, 18^. Various kinds of stretchable electronic devices using buckled structures have been reported as their mechanical properties can be easily modified by controlling the period and height of the single-buckled structure^19, 20^. However, in conventional buckled structures, stretchability of the buckled device is ensured only along a single axis and is mostly dependent on the stretching conditions of the same axis. Therefore, a new approach is needed to improve the performance of stretchable electronic devices using the established buckled structures.

In this study, we developed cross-buckled structures that can overcome the abovementioned limitations of conventional buckled structures, for the realization of stretchable electronics. The cross-buckled structure with orthogonally positioned buckled ribbons is a reliable structure even under multi-directional strains and can be fabricated using simple transfer printing process. We investigate the effect of compressive strain on the cross-buckled structures for the first time by testing the reliabilities of single- and cross-buckled structures under compressive strain, as opposed to previously reported studies, which mostly focused on deformation along a single-axis during stretching. The easily amendable size and spacing of the cross-buckled structure can lead to the manufacture of diverse semiconductors as stretchable electronic devices in textile patterns. Stretchable textile electronics based on cross-buckled structures will have a significant impact on various high-performance applications demanding stretchability.

## Results and Discussion

**Figure 1:**
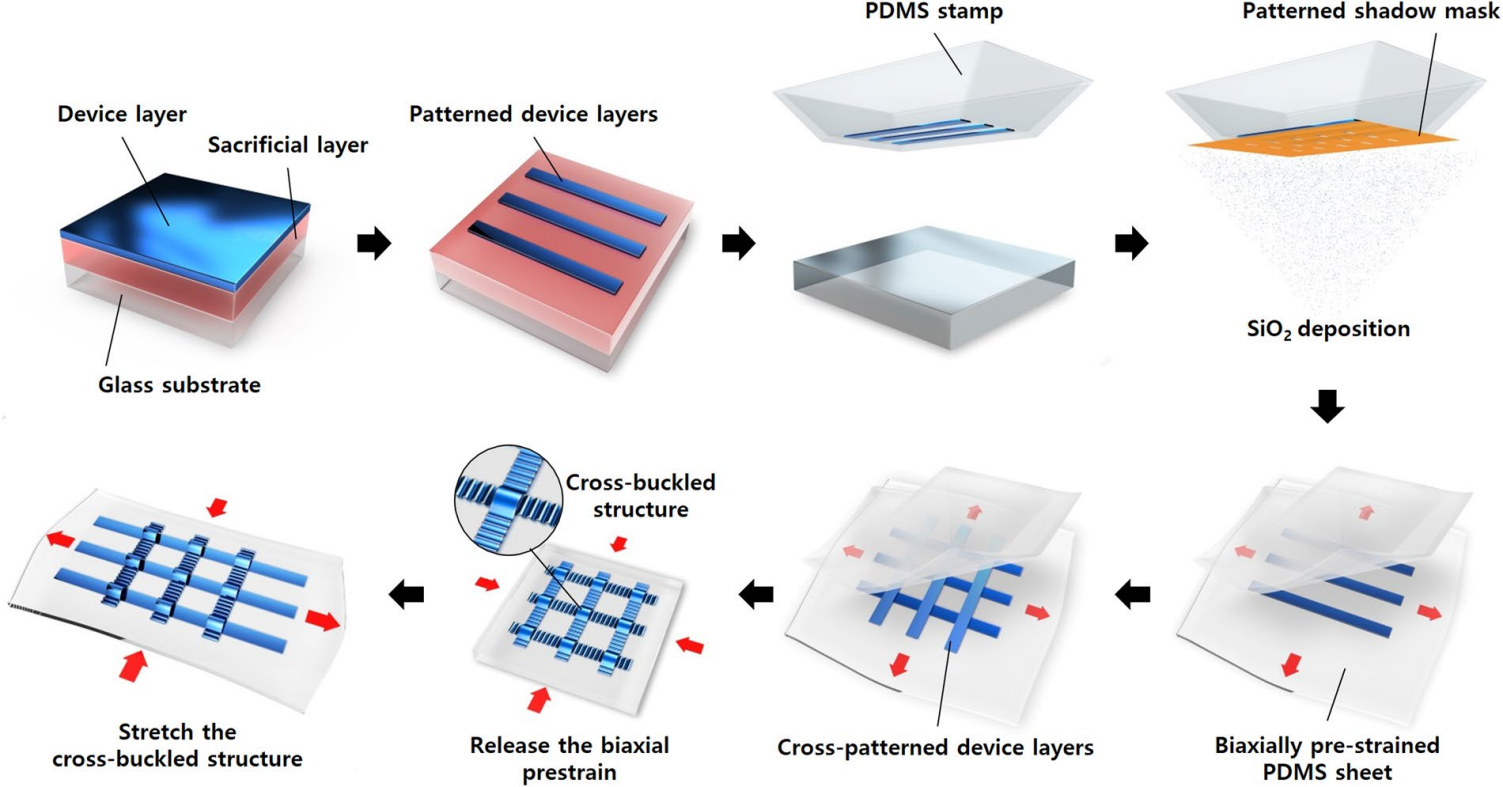
Schematic illustration of the procedure for the fabrication of cross-buckled structures.

Figure 1 shows the schematic diagram of the procedure for the fabrication of cross-buckled structures. The device layers, including the metal electrode and the semiconductor, were fabricated and patterned on a pre-deposited sacrificial layer which was dissolved later by a selective etchant to detach the device layer from the glass substrate. Using polydimethylsiloxane (PDMS) stamp as the transferring carrier, the device layer was detached from the glass substrate^21^. Before transferring the device layer to the stretchable substrate, silicon dioxide (SiO_2_) was deposited on the bottom of the detached device layer through the patterned shadow mask. The patterened SiO_2_ acts as a functional group for the formation of chemical bonds with the stretchable PDMS substrate^22^. By controlling the number of intervals between the chemical bonds, buckles with different periods and heights can be formed. Cross-patterned device layers were fabricated by repeating the transfer printing process of the device layers on the biaxially pre-strained substrate. After the transfer printing process, the cross-buckled structure was obtained by releasing the biaxial prestrains.

**Figure 2:**
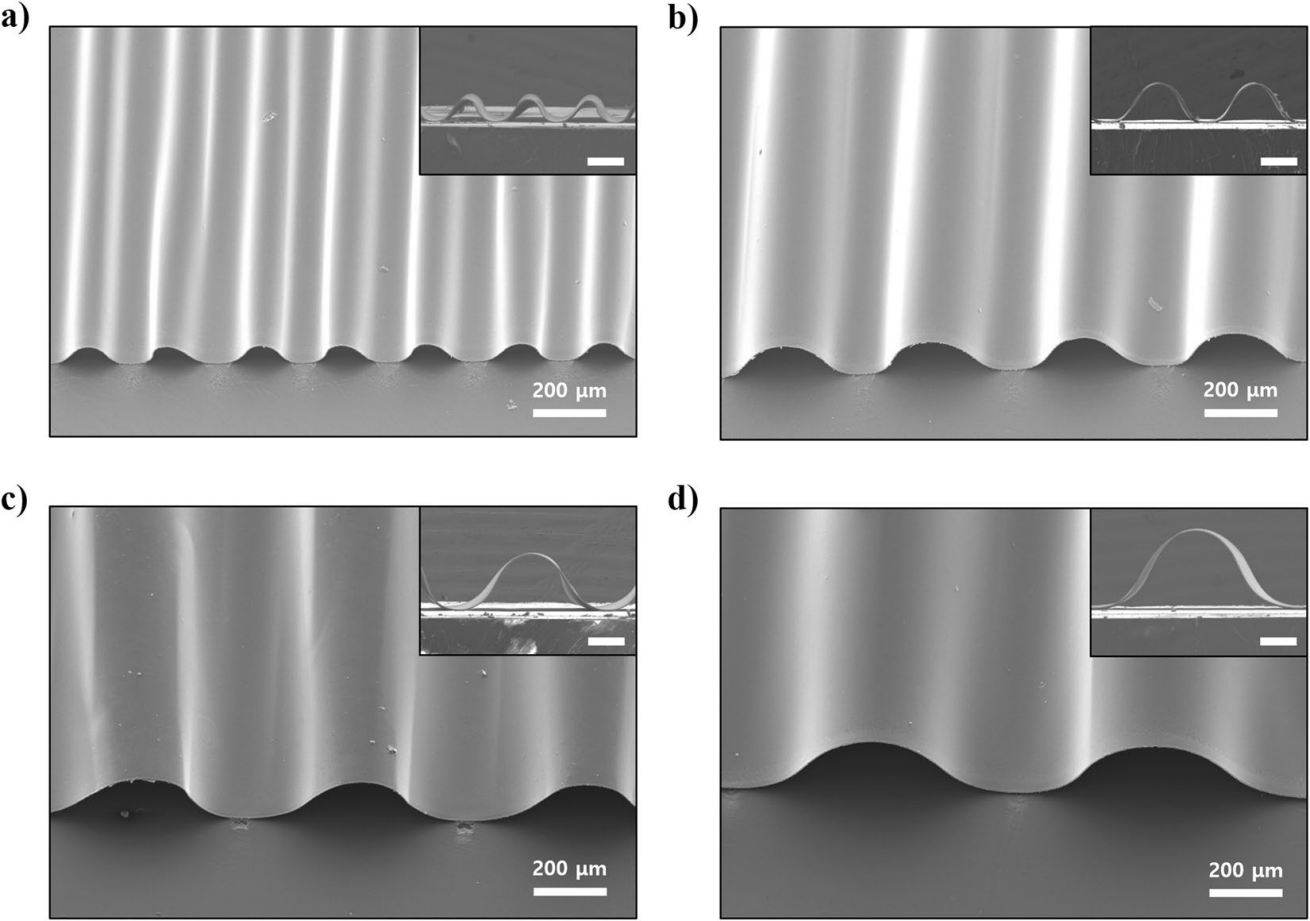
SEM images of the buckled aluminum electrode. Buckled aluminum electrode with chemical bonding intervals of (**a**) 200 μm, (**b**) 400 μm, (**c**) 600 μm, and (**d**) 800 μm. The insets show the cross-sectional SEM images of the buckled aluminum electrodes with 150 μm of scale bar.

Controlling the period and the height of the buckled structure is crucial for the fabrication of the cross-buckled structure because the upper buckled ribbon at the intersectional area of the cross-buckled structure should have no contact with the bottom buckled ribbon. First, controlling the period and the height of the single-buckled structure were verified by fabricating buckled aluminum electrode which is a widely used metal electrode for electrical interconnections. In general, the period and the height of buckled structures are affected by various parameters such as amount of prestrain, mechanical properties of the material, thickness of the material, and intervals of each chemical bond^14, 19, 23, 24^. Among them, controlling the intervals between each chemical bond is the easiest approach to develop a particular configuration of the buckled structure. Figure 2 shows the tilted and the cross-sectional scanning electron microscope (SEM) images of the buckled aluminum electrode. Figure 2a–d shows the formation of the buckled structure at chemical bonding intervals of 200, 400, 600, and 800 μm respectively. As shown in the images in the inset, broadening the interval between each chemical bond resulted in proportional increases in the period and the height of the buckles. This result indicates the possibility of fabricating cross-buckled structures by tuning the specific period and height of the buckles.

**Figure 3:**
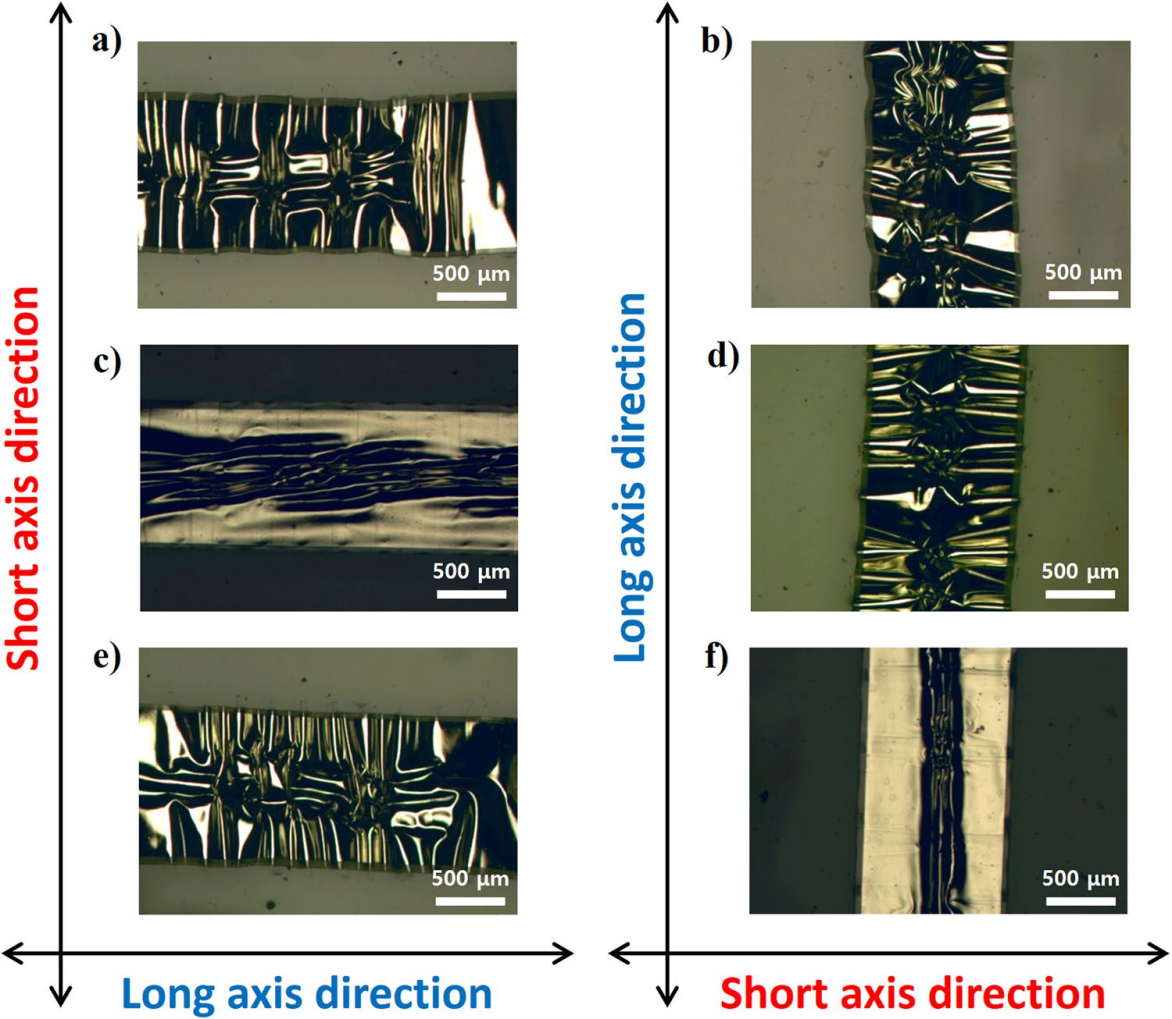
Optical images of cross-buckled aluminum electrodes (**a**,**b**) before stretching, (**c**,**d**) after stretching in x-axis direction, and (**e**,**f**) after stretching in y-axis direction.

As a first demonstration of the cross-buckled structure, we manufactured cross-buckled aluminum electrodes by a simple transfer printing process. Contrary to conventional buckled structures, prestrains were applied biaxially on the stretchable substrate. Therefore, the configuration of the cross-buckled structure appeared quite different from those of conventional buckled structures. Figure 3 shows the optical microscopic (OM) images of the cross-buckled aluminum electrode. Figure 3a,b shows the optical images of the surface of the cross-buckled aluminum electrode after the removal of the biaxial prestrains. In the middle of each buckled electrode, a squeezed buckled structure, which is a mixture of buckles oriented along x- and y-axes, was formed by the release of the biaxial prestrains and led to a multi-directional irregular buckled structure that deforms freely under multi-directional strains. Major buckles are formed by ‘long axis’ directional prestrain which has the same orientation as that of the buckled electrode, and minor buckles are formed mostly in the middle of the buckled electrode by ‘short axis’ directional prestrain which has a vertical orientation with that of the buckled electrode.

Figure 3c,d represents the surface images of the cross-buckled aluminum electrode during x-axis directional stretching. The single-axis stretching strain resulted in simultaneous orthogonal compressive strain^25^. The amount of the tensile strain is proportionally asymmetrical compared to that of the compressive strain, which induces minor deformations to the cross-buckled aluminum electrode. In Fig. 3c, the ‘long axis’ of buckled structure is parallel to the stretching direction. Therefore, the major buckles are stretched by the external strain whereas the minor buckles are compressed in the y-axis direction. The x-axis directional buckles are the major buckles in Fig. 3c; therefore, the deformation of the buckles is noticeable. On the contrary, the ‘long axis’ of buckled structure is orthogonal to the stretching direction in Fig. 3d. Hence, the minor buckles are stretched by the external strain whereas the major buckles are compressed in the y-axis direction. Therefore, the deformation of the buckles during the x-axis directional stretching is insignificant, because the y-axis directional buckles are the major buckles. Figure 3e,f represents the surface images of the cross-buckled aluminum electrode during the y-axis directional stretching. As the direction of stretching is orthogonal with that of abovementioned cross-buckled electrodes, the deformation of buckled structure is opposite to Fig. 3c,d. In Fig. 3e, the ‘long axis’ of buckled structure is orthogonal to the stretching direction, the minor buckles are stretched in the y-axis direction. On the contrary, the ‘long axis’ of buckled structure is parallel to the stretching direction in Fig. 3f, the major buckles are stretched in the y-axis direction. Therefore, the cross-buckled aluminum electrode is freely deformable under the external tensile and the compressive strains because of the squeezed buckled structures.

**Figure 4:**
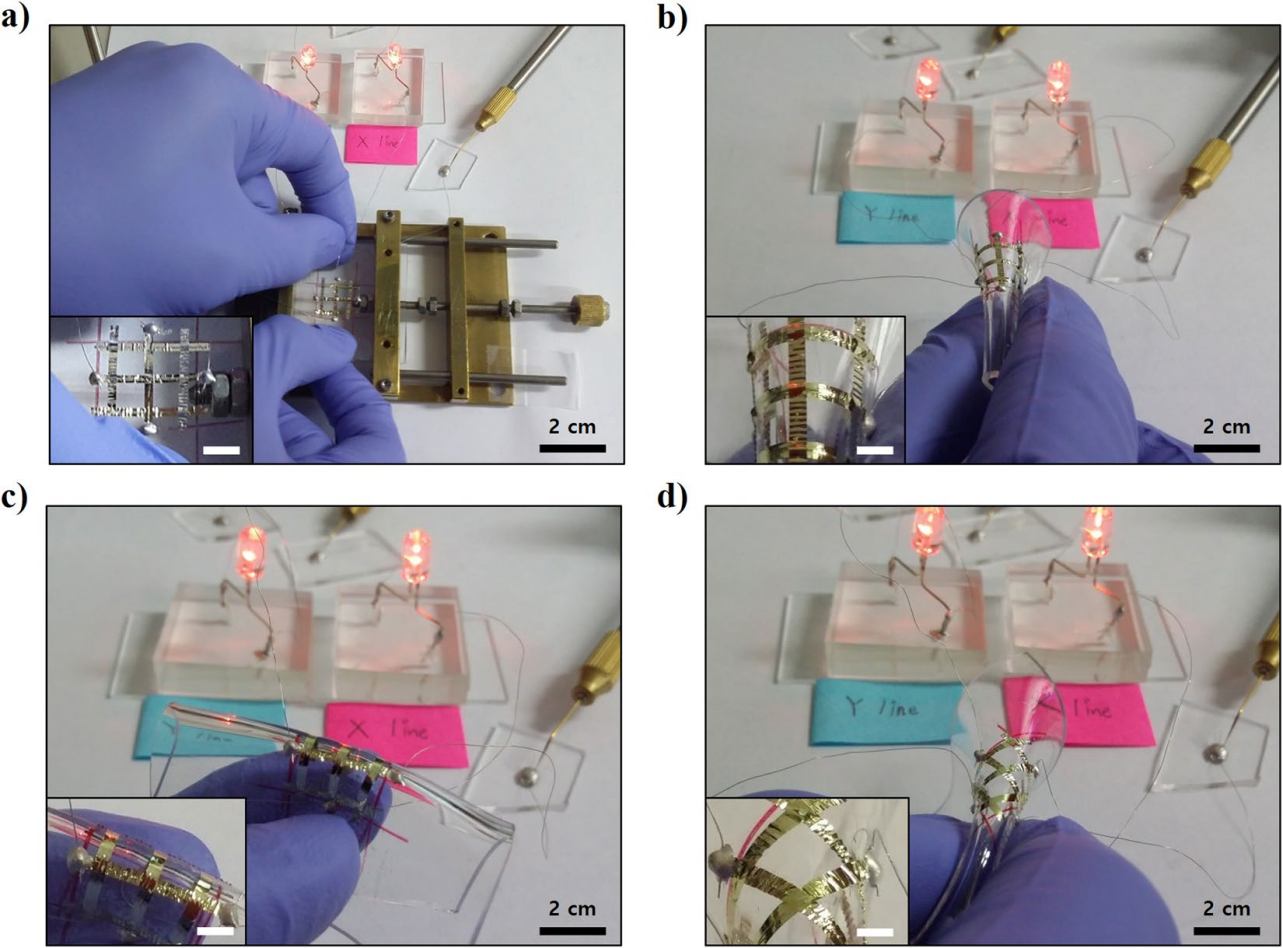
Photographs of LEDs connected to the cross-buckled aluminum electrodes. LEDs in the left and right side were connected to the cross-buckled aluminum electrode in the vertical and horizontal directions, respectively. Continuous LED lighting observed during (**a**) biaxial stretching and bending in the (**b**) y-axis, (**c**) x-axis, and (**d**) diagonal directions.

The electrical reliability of the cross-buckled aluminum electrode was confirmed by a lighting test using light emitting diodes (LEDs). Figure 4 shows the setup used for testing the electrical reliability of the cross-buckled aluminum electrode under various stretching and bending tests. The two LEDs were individually connected to the horizontal and vertical buckled aluminum electrodes to verify the electrical characteristics under multi-directional strains. The LEDs that were connected to each cross-buckled aluminum electrode showed continuous lighting under biaxial stretching (Fig. 4a). Figure 4b–d shows the electrical reliability of the electrode during the bending tests along y-axis, x-axis, and diagonal directions, respectively. Even after folding the device, the LED showed stable lighting in all the bending tests. The diverse measurements carried out for the single-buckled structure and the cross-buckled structure indicate the possibility of fabrication of stretchable electronic devices using cross-buckled structures.

**Figure 5:**
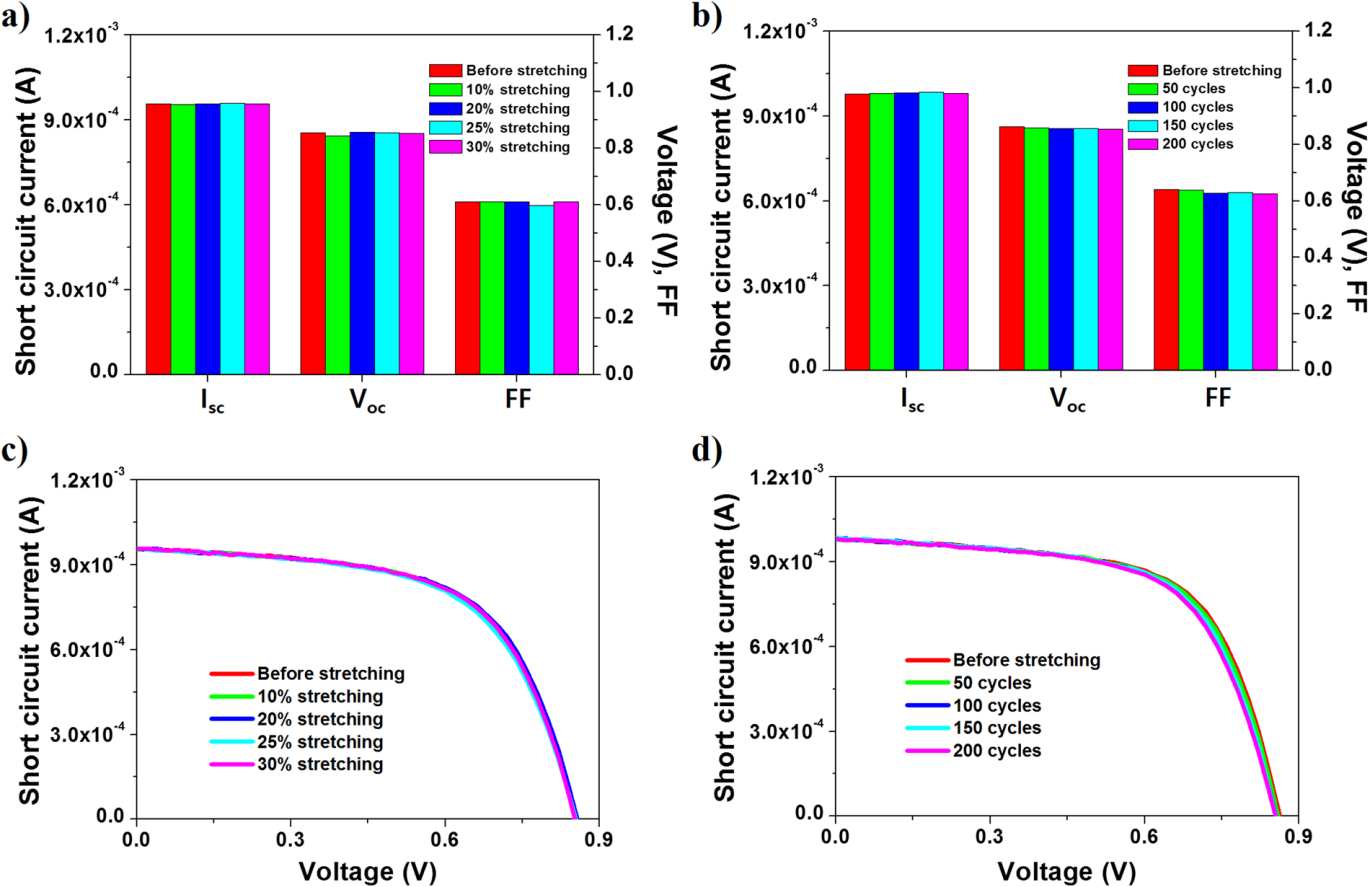
Photovoltaic characteristics of single buckled ribbon as a function of (**a**) tensile strain and (**b**) stretching cycle. Current-voltage curves of single buckled ribbon as a function of (**c**) tensile strain and (**d**) stretching cycle.

In order to apply the cross-buckled structure to conventional electronic devices, we fabricated stretchable thin film silicon solar cells using the cross-buckled structure. The thin film silicon solar cell was fabricated on the pre-deposited sacrificial layer and transfer printed using the same fabrication processes as those of the cross-buckled aluminum electrode. Each buckled ribbon represents a single solar cell and the properties were tested under stretching condition. Figure 5 shows the variations in the photovoltaic characteristics at different tensile strains and stretching cycles. From 0 to 30% of the external tensile strain, there was no noticeable degradation in the short circuit current (I_sc_), the open circuit voltage (V_oc_), and the fill factor (FF) (Fig. 5a,c). In addition, the cross-buckled thin film silicon solar cells showed stable photovoltaic characteristics for about 200 stretching cycles (Fig. 5b,d). Thus, the cross-buckled structure developed for stretchable electronic devices was successfully adapted to thin film silicon solar cells while maintaining the photovoltaic properties during stretching.

**Figure 6:**
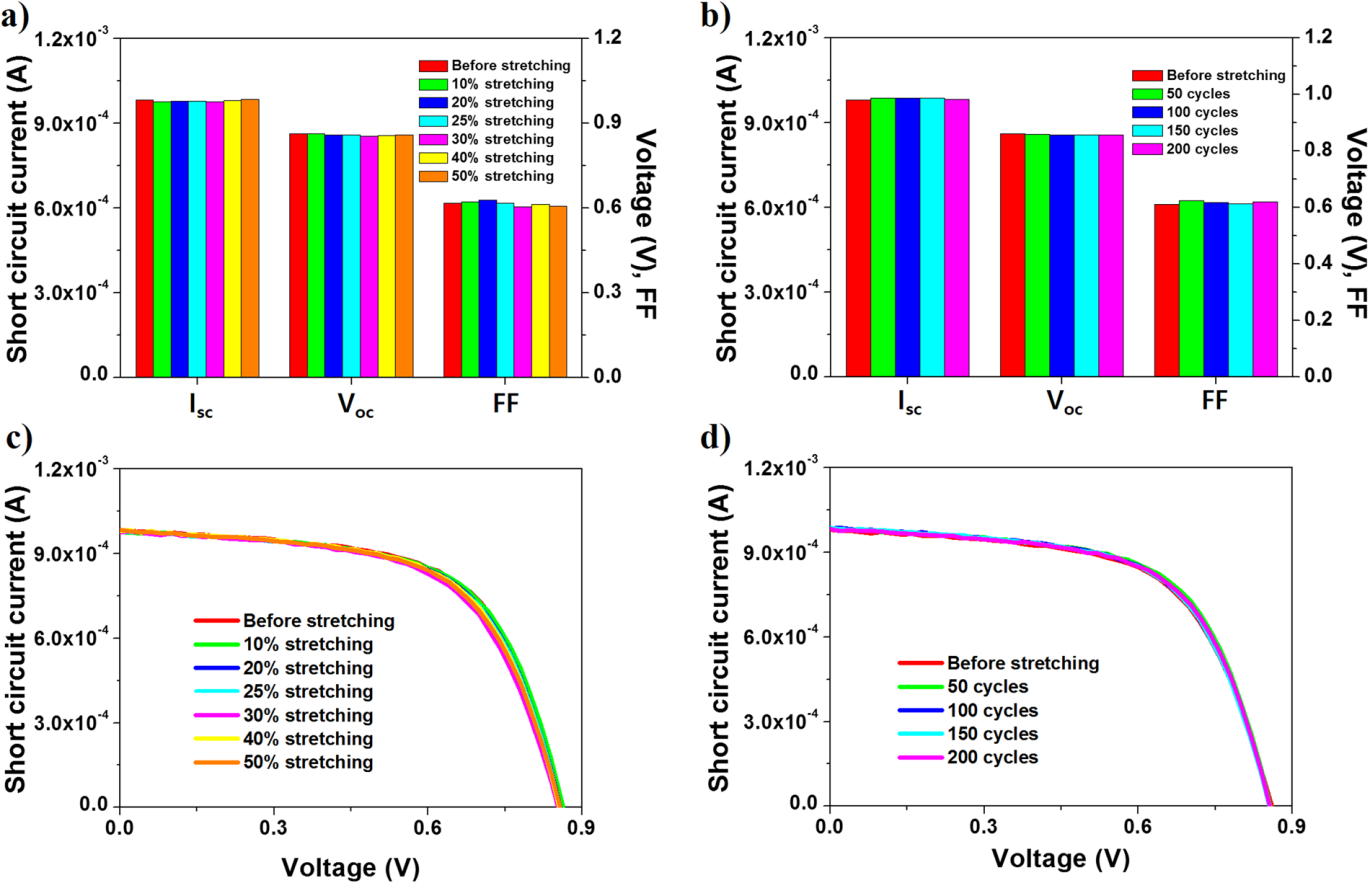
Photovoltaic characteristics of single buckled ribbon as a function of (**a**) tensile strain that is orthogonal to the direction of the compressive strain and (**b**) stretching cycle. Current-voltage curves of single buckled ribbon as a function of the (**c**) tensile strain that is orthogonal to the direction of the compressive strain and (**d**) stretching cycle.

As mentioned before, the application of single-axis directional tensile strain to the stretchable substrate resulted in orthogonal compressive strain. Thus, we also demonstrated the stability of the cross-buckled thin film silicon solar cells under compressing for the first time, whereas most of the studies on buckled structures are focused on the configuration and the properties during stretching. Figure 6 shows the variation in the photovoltaic characteristics under compressing. To verify the properties of the cross-buckled thin film silicon solar cell under compressing, an external tensile strain that is orthogonal to the direction of the compressive strain was applied. In the compressed state, the cross-buckled thin film silicon solar cell maintained its properties up to 50% of the external tensile strain (Fig. 6a,c) and 200 cycles of stretching (Fig. 6b,d). The increase in the induced compressive strain by the orthogonal tensile strain is inequivalent to the increase in the tensile strain^25^. The deformation of the cross-buckled thin film silicon solar cell under the compressive strain was found to be much less than that under the tensile strain. Therefore, the characteristics of the cross-buckled thin film silicon solar cell were stable even under the compressive strain caused by the application of 50% of the tensile strain.

**Figure 7:**
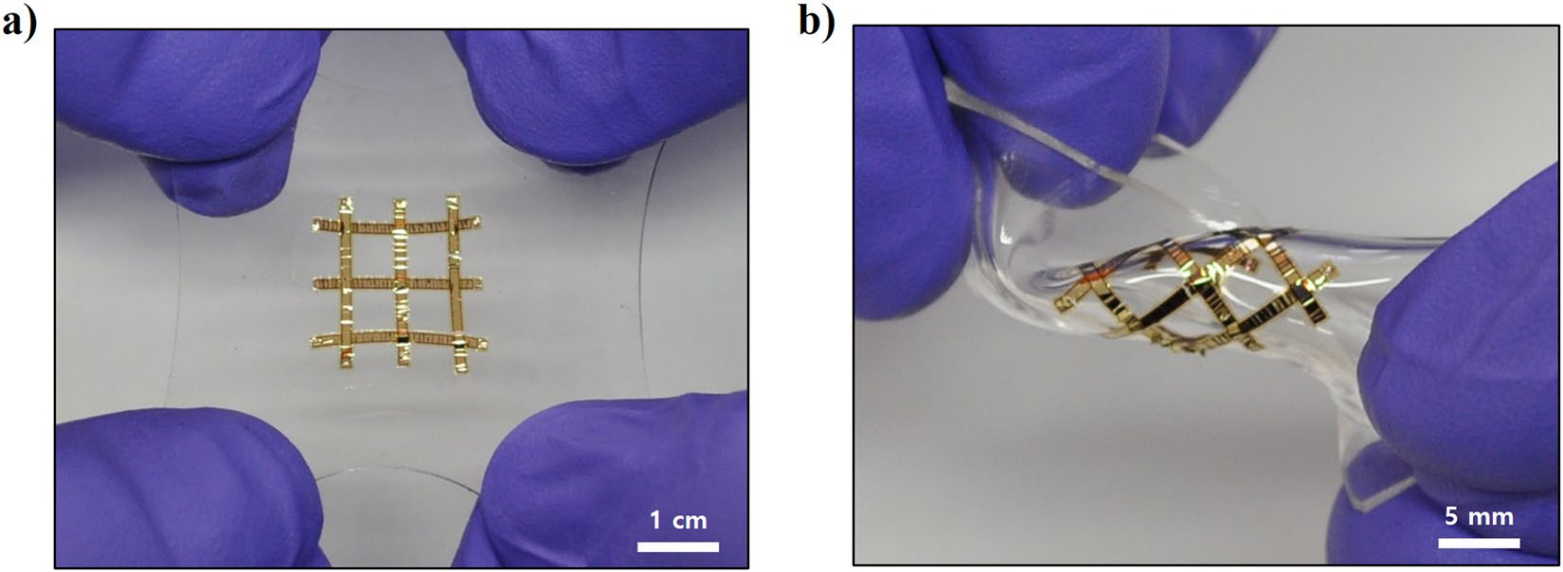
Photographs of the cross-buckled thin film solar cells. Cross-buckled thin film solar cells under (**a**) biaxially stretching state and (**b**) twisting state.

Figure 7 shows the various configurations of the cross-buckled thin film silicon solar cells under twisting and stretching. Figure 7a shows 6 lines of the transfer printed thin film silicon solar cells in the cross-buckled structure. During the simultaneous application of the tensile strain in four different directions, most of the buckles were stretched following the external tensile strain. With stable mechanical characteristics under multi-directional strains, the cross-buckled thin film silicon solar cells can endure twisting conditions (Fig. 7b).

In this study, although we demonstrated only 6 lines of cross-buckled thin film silicon solar cells on a laboratory scale, the simple and optimized fabrication process can be expanded to produce textile structures. In addition, the thin film silicon solar cell is a promising candidate for applications such as portable power supply for wearable electronics. Thus, the textile thin film silicon solar cells with the cross-buckled structure can function as portable power supplies by attaching them to the surfaces of fabrics or human skin.

## Conclusion

We developed cross-buckled structures of thin film silicon solar cells that exhibit reliable mechanical and electrical characteristics under multi-directional tensile and compressive strains. The properties of the cross-buckled thin film silicon solar cells were stable under various types of deformation including twisting, bending, folding, and stretching, whereas conventional buckled structures exhibit limited stretchability only in single-axis direction. As demonstrated in multi-directional stretching, the properties of the cross-buckled structure were also verified under the compressive strain induced by orthogonal stretching. We employed simple transfer printing process and biaxial prestrain to fabricate the cross-buckled structure. This technique can be easily modified and adapted to the desired configuration of stretchable electronic devices by controlling the intervals of each chemical bond. We also demonstrated that the cross-buckled thin film silicon solar cells can be fabricated in textile pattern through optimized repetitive transfer printing process, thus broadening the scope of the cross-buckled structures. The stretchable cross-buckled structures can be applied to various stretchable electronic devices without the need for optimization of complex processes to meet the target configuration. This attempt to manufacture new type of buckled structures to realize stretchability encourages future developments in the area of stretchable electronics.

## Methods

### Fabrication of sacrificial layer

Conventional soda-lime glass was cleaned with acetone, isopropyl alcohol, and deionized water in a sonication bath. A 100 nm thick germanium layer was deposited on the cleaned glass substrate by electron-beam evaporation. The germanium oxide layer was formed by dry oxidation of germanium at 510 °C for 45 min using a conventional furnace.

### Deposition of aluminum electrode and thin film silicon solar cells

Uniform layers of thin film silicon solar cells were deposited by cluster-type plasma enhanced chemical vapor deposition (PECVD) and sputtering. First, a 500 nm thick aluminum doped zinc oxide (AZO) layer, as the bottom electrode, was deposited on the substrate at the substrate temperature of 200 °C by sputtering. The p-type, the n-type, and the intrinsic amorphous silicon layers were deposited at thicknesses of 13, 25.7, and 460 nm, respectively, at 250 °C by PECVD. Subsequently, a 300 nm thick AZO layer, as the top contact layer, was deposited on the thin film silicon layers by sputtering at the substrate temperature of 200 °C. Finally, a 100 nm thick aluminum layer, as the top electrode, was deposited on the top AZO layer by thermal evaporation.

### Fabrication of single-buckled aluminum electrode

The aluminum electrode was patterned by the lift-off technique. Polyimide was spin-coated on top of the aluminum electrode to encapsulate all the device layers, and was patterned by reactive ion etching (RIE). Encapsulated aluminum electrode was separated from the substrate by etching the sacrificial layer with deionized water using the PDMS stamp as the transferring holder. SiO_2_ was deposited on the bottom of the aluminum electrode using a patterned shadow mask to form localized chemical bonds. The aluminum electrode was transfer-printed onto the prestrained PDMS substrate. By releasing the prestrain, the stretchable PDMS substrate was elastically relaxed back to its original shape, and the areas of the aluminum electrode between the localized chemical bonds popped up, forming the buckled structure at specific intervals.

### Fabrication of cross-buckled thin film silicon solar cell

The top and bottom AZO layers were etched with dilute hydrochloric acid and the thin film silicon layers were etched by RIE. Polyimide was spin-coated on top of the thin film silicon solar cells to encapsulate all the device layers, and then patterned by the RIE. The width and length of a single solar cell ribbon are 1.24 mm and 14.46 mm, respectively. The thin film silicon solar cells were separated from the substrate by same transfer printing process with buckled aluminum electrode. All the thin film silicon solar cells were transfer-printed onto the biaxially prestrained substrate in the shape of textile using the PDMS stamp. By releasing the biaxial prestrain, the squeezed buckled structure was positioned in the middle of the buckles by biaxial compressive strains, and large buckles were formed in the intersectional area of the cross-buckled thin film silicon solar cells due to the absence of chemical bonds.

### Characterization of devices

The properties of the solar cell were determined under AM 1.5 G illumination (solar simulator, Model Sol2A, Oriel) at 25 °C. The cross-buckled thin film silicon solar cells were subjected to tensile and compressive strains using a stretching machine in order to verify the mechanical and electrical reliability.
